# Supplementary material for: Two-Month Voluntary Ethanol Consumption Promotes Mild Neuroinflammation in the Cerebellum but Not in the Prefrontal Cortex, Hippocampus, or Striatum of Mice
Source: Int J Mol Sci. 2024 Apr 10;25(8):4173. doi: 10.3390/ijms25084173 (PMC11050159; doi:10.3390/ijms25084173)
Supplement: Supplementary file 1 [file ijms-25-04173-s001.zip › ijms-2920689-supplementary.pdf]

SUPPLEMENTARY TABLE 1

Gene	Forward primer	Reverse primer	Efficiency
MCP1	5'-CCAGCAAGATGATCCCAATG-3'	5'-TCTGGACCCATTCCTTCTTG-3'	0.942
GAPDH	5'-TGGAGAAACCTGCCAAGTATGA-3'	5'-TGGAAGAATGGGAGTTGCTGT-3'	1.000
Iba1	5'-CCGAGGAGACGTTCAGCTAC-3'	5'-GACATCCACCTCCAATCAGG-3'	0.965
IL6	5'-TTCCATCCAGTTGCCTTCTTG-3'	5'-TATCCTCTGTGAAGTCTCCTCTC-3'	0.984
IL-1β	5'-TCGCTCAGGGTCACAAGAAA-3'	5'-CATCAGAGGCAAGGAGGAAAAC-3'	0.941
TNFα	5'-CATCTTCTCAAAATTCGAGTGACAA-3'	5'-TGGGAGTAGACAAGGTACAACCC-3'	0.933
COX2	5'-GAAGTCTTTGGTCTGGTGCCT-3'	5'-GCTCCTGCTTGAGTATGTCG-3'	0.970
TLR4	5'-TCAGAACTTCAGTGGCTGGA-3'	5'-AGAGGTGGTGTAAGCCATGC-3'	0.971
CCR2	5'-GTGTACATAGCAACAAGCCTCAAAG-3'	5'-CCCCCACATAGGGATCATGA-3'	0.946
MIP1α	5'-ATATGGAGCTGACACCCCGA-3'	5'-TCAACGATGAATTGGCGTGG-3'	0.963
MCP5	5'-TATTGGCTGGACCAGATGCGG -3'	5'-ACACTGGCTGCTTGTGATTCT-3'	0.965
CXCL2	5'-CCCAGACAGAAGTCATAGCCAC-3'	5'-TGGTTCTTCCGTTGAGGGAC-3'	0.966

Supplementary Table 1. Forward and reverse primers used in this study. Efficiency was calculated using the following equation: E = -1+10(-1/slope), where the slope was obtained from the linear regression of Ct v/s log[DNA] using four 10-fold dilutions of cDNA obtained from the PCR product of the experiments conducted in the study. Assays included PCR product dilutions from 1/1.000.000 to 1/1.000.000.000, prepared in serial dilutions, and assayed in duplicate.

SUPPLEMENTARY TABLE 2

Name	Sequence		
miLinker	pp(r)A.GGCCGAACTACGACCTGCATAAACGG.ddC		
miQRT	5'- CCCAGTTATGGCCGTTTATGCAGGT-3'		
Upm2a	5'- CCCAGTTATGGCCGTTTA-3'		
**Gene**	**Forward primer**	**Reverse primer**	**Efficiency**
mmu-miR-21a-5p	5'-GCTTATCAGACTGATGTTGAGGC-3'	Upm2a	0.991
mmu-miR-146a-5p	5'-GAGAACTGAATTCCATGGGTTG-3'	Upm2a	0.995
mmu-miR-155-5p	5'-TTAATGCTAATTGTGATAGGGGTG-3'	Upm2a	0.945
mmu-Let-7d-5p	5'-AGAGGTAGTAGGTTGCATAGTTG-3'	Upm2a	0.984
RNU6	5'-GCAAGGATGACACGCAAATT-3'	Upm2a	1.000

Supplementary Table 2. MiQPCR sequences and primers used in this study. Efficiency was calculated using the following equation: E = -1+10(-1/slope), where the slope was obtained from the linear regression of Ct v/s log[DNA] using four 10-fold dilutions of cDNA obtained from the PCR product of the experiments conducted in the study. Assays included PCR product dilutions from 1/1.000.000 to 1/1.000.000.000, prepared in serial dilutions, and assayed in duplicate.
